# Predicting microscopic vehicle collision risks at toll plaza diverging area using bayesian dynamic logistic regressions

**DOI:** 10.1371/journal.pone.0332929

**Published:** 2025-10-27

**Authors:** Xi Li, Yi Fei, Kongning Jin, Lu Xing, Yujie Zhang, Fengwei Yang, Shuyu He

**Affiliations:** 1 School of Traffic and Transportation Engineering, Changsha University of Science and Technology, Changsha, Hunan, PR China; 2 Hunan Key Laboratory of Smart Roadway and Cooperative Vehicle-infrastructure Systems, Changsha University of Science and Technology, Changsha, Hunan, PR China; 3 School of Automation, Central South University, Changsha, Hunan, PR China; University of Wisconsin-Milwaukee, UNITED STATES OF AMERICA

## Abstract

The absence of lane markings at toll plaza diverging areas results in frequent vehicle weaving motions, making these areas typical high-risk bottlenecks on highways. Existing conflict prediction methods often rely on historical data and static models, which lack adaptability to dynamic changing traffic conditions. This study proposes a Bayesian dynamic logistic regression approach capable of self-adaptive prediction of vehicle collision risks at toll plaza diverging areas. First, the aggregated traffic characteristics were extracted from the high-precision vehicle trajectory data and the indicator Extended Time-to-Collision (ETTC) was employed to measure multi-directional vehicle collision risks. Then, Bayesian dynamic logistic regression models were developed based on aggregated traffic characteristics from different sampling strategies. Results show that as the data volume increases, the Area Under the Curve (AUC) values of these models all gradually exceeds 0.9, demonstrating strong self-adaptive correction capabilities. Compared with standard logistic regression models, the Bayesian dynamic logistic regression models identified more influencing factors and required only 20% of the data for initialization, while continuously updating estimates with incoming data, significantly reducing computational resource demands for collision risk prediction. Furthermore, sensitivity analysis of the forgetting parameter indicates that incorporating richer prior information enhances predictive accuracy. These findings provide valuable insights for developing tailored management strategies to reduce potential traffic conflicts at toll plaza diverging areas.

## Introduction

Toll plazas serve as critical parts for both traffic control and toll collection, playing a key role in supporting the efficient operation of highways [1,2]. In many Asian countries, such as China, Japan, South Korea, and India, the forms of the toll plazas are still dominant by the Traditional Mainline Toll Plazas (TMTPs) [3,4].

**Fig 1:**
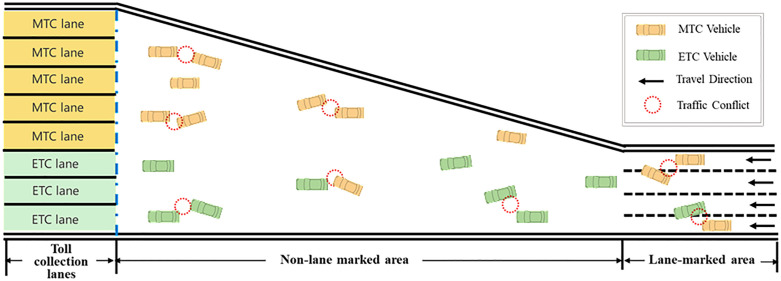
Potential vehicle conflicts at the TMTP diverging area.

The diverging areas of TMTPs are gradually widening transition areas that connect the highway mainlines to individual toll lanes [5]. One of the most notable features of this area is the absence of lane markings or physical separation between Electronic Toll Collection (ETC) lanes and Manual Toll Collection (MTC) lanes. Fig 1 presents a schematic diagram of a typical diverging area in a TMTP. ETC lanes are generally arranged on the inner side, while MTC lanes are on the outer side. ETC and MTC vehicles are required to rapidly decelerate and select their matching toll lanes within a limited distance, resulting in frequent weaving motions and speed variations [6–8]. Moreover, this complexity is further compounded by the speed differential between vehicle types that ETC vehicles can pass through the toll lanes at 20–30 km/h without stopping, while MTC vehicles must decelerate to a full stop for manual payment. The lack of lateral constraints and significant speed variations lead to frequent conflicts between vehicles at arbitrary angles, making the TMTP a high-risk area. Therefore, accurately predicting the collision risks in the diverging area is essential for developing effective traffic management strategies and enhancing highway safety performance.

Early safety prediction primarily relied on historical crash data. However, historical crash data has significant limitations, as it requires a long period of observation and are prone to underreporting, underestimating crash risks at certain places [9,10]. With advancements in data collection technologies, high-precision data, such as vehicle trajectory data and image data, have increasingly been utilized for traffic safety analysis from a microscopic perspective. Using trajectory data to predict traffic collision risks between traffic participations, as a proactive safety prediction method, has gained considerable attention [1,11]. Unlike crash data, conflict data can be captured in large quantities over short observation periods and under normal traffic conditions, making it more suitable for timely risk identification and intervention. However, the large volume of trajectory data, coupled with numerous features and redundant information, poses challenges for its application in safety assessments, particularly for real-time traffic conflict prediction [12].

To address this issue, this study utilizes the aggregated time series traffic characteristics extracted from vehicle trajectory data to predict their conflicts. Compared to directly using raw trajectory data, aggregated traffic characteristics offer several advantages. First, aggregated traffic characteristics filter out redundant information while preserving key features, enhancing both the training efficiency and generalization ability of the model. Second, they reveal the relationships between traffic flow features, environmental factors, and collision risks, which is particularly valuable for safety management in complex traffic areas such as the diverging areas [13,14].

The high collision risk is not only related to the complex structure of toll plaza diverging areas, but also to the frequent changes of traffic conditions. For example, the safety condition in toll plazas often greatly fluctuates for various reasons, such as incidents and traffic volume changes. Therefore, considering dynamic changes of influencing factors is crucial for improving the prediction accuracy of vehicle collision risks. Base on the advantages of aggregated time series traffic characteristics, this study integrates a Bayesian updating approach to dynamically predict vehicle collision risks in toll plaza diverging areas. The Bayesian updating approach are proven to have the ability of integrating prior information and continuously update results by incorporating new data to realize parameters’ self-adoptive update, adapting to traffic condition changes across different times and locations [15,16]. While dynamic Bayesian models have been applied in previous studies to estimate and predict traffic conflicts and crash risks on structured road segments [17–19], their effectiveness in highly dynamic, non-lane-based complex traffic environments remains to be further explored.

Therefore, this study aims to optimize vehicle collision risk prediction at toll plaza diverging areas by replacing traditional lagged predictions based on historical data with a dynamic framework capable of continuously updating predictions with new data. The rest of this study is organized as follows. Previous studies about vehicle collision risk prediction are reviewed in the Literature review section. The methodology about surrogate safety measurements and Bayesian dynamic logistic regression model is introduced in the Methodology section. The data collection and procedures are presented in the Data section. The Result and discussions section discusses the model results and the conclusions are summarized in the Conclusion section.

## Literature review

### Data sources for vehicle collision risk evaluation

Previous studies about the influencing mechanism of vehicle collision risk mainly used the data collected from historical crash [20,21] and simulation data [6,22,23]. For example, Zhao and Lee [24] used the crash data conducted on Gardiner Expressway in Toronto to evaluate the rear-end collision risk of cars and heavy vehicles. Sha [23] utilized the Simultaneous Perturbation Stochastic Approximation (SPSA) optimization algorithm to calibrate multiple parameters of driving models and reproduced the actual vehicle conflict distribution using the simulation software Simulation of Urban Mobility (SUMO). Although historical crash data and simulation data have made a great contribution to vehicle collision risk evaluation, there are still some limitations. As discussed above, historical crash data may lead to biased safety assessments, as they require a long observation period to accumulate sufficient data for analysis and are often subject to underreporting or misreporting [25,26]. Simulation data often rely on oversimplified behavioral assumptions, such as same driver reactions or idealized vehicle dynamics, making it difficult to fully capture the heterogeneity of real-world driving behaviors [27]. In contrast, vehicle trajectory data can offer rich spatiotemporal information that reflects the subtle variations in driver behavior across time, space, and traffic conditions. This allows for a more precise and proactive evaluation of collision risks, particularly in complex, non-lane-based environments.

With the development of traffic conflict techniques, conflict data becomes an alternative in evaluating and predicting vehicle collision risks [28,29]. Vehicle conflicts data can be used to analyze the effects of vehicles’ microscopic moving status on collision risks, unlike crash data only judging whether there is a crash or not. Traffic conflict techniques apply various conflict indicators to calculate potential risks, such as Time-to-Collision (TTC), Deceleration Rate to Avoid the Crash (DRAC) and others [24,30,31]. Once the indicator value reaches to the preset threshold, the potential vehicle conflict will be identified. Vehicle trajectory data and its derived aggregated time series traffic characteristics have become one of the major data sources for exploring vehicle conflicts due to its microscopic characteristics [32–35]. Potential conflicts extracted from trajectory data can greatly shorten the data collection time, which overcomes the limitation of insufficient quantity of historical crash data, thereby improving the efficiency of collision risk evaluation. For example, Torkashvand [36] assessed rear-end collision risks on two-lane roads using a dynamic probabilistic risk approach, evaluating the influence of overtaking behavior on the Time-to-Collision (TTC) threshold. An [37] developed a rear-end collision prediction model for congested highway segments using vehicle trajectory data and a Gated Recurrent Unit (GRU)-based end-to-end model. Based on vehicle trajectory data extracted from Unmanned Aerial Vehicle videos, Chen [31] compared the performance of three conflict indicators, TTC, DRAC, and the Absolute value of the Derivative of Instantaneous Acceleration (ADIA), in real-time rear-end collision risk prediction on highways.

### Modeling approaches for collision risk prediction

The collision risk prediction models can be generally divided into parametric models and non-parametric models. Both models have been widely used in predicting vehicle collision risk with different research objectives, traffic scenarios and datasets. Parametric models, such as logistic regression [38], random effect logit models [11,39], negative binomial models [40], and Bayesian related models [17,41], are well-suited for explaining the relationships between factors and vehicle collision risks [40]. However, they require the data distribution must meet the assumption, otherwise the model may produce wrong inference. In contrast, non-parametric models, like Support Vector Machine (SVM) (Dong et al., 2015), Random Forest [42], and Neural Networks [43], offer greater flexibility, as they do not impose distributional assumptions and can model complex, non-linear relationships. While non-parametric models generally achieve higher prediction accuracy, they lack interpretability compared to their parametric counterparts [39,44].

Besides, some methods consider the temporal transferability of collision risk predictions, thereby enhancing model generalization. Traffic data are usually collected in a sequential manner and often exhibit temporal dependencies. These models use time-series data to capture the spatiotemporal evolution of traffic collision risks, such as Long Short-Term Memory (LSTM) networks and Bayesian updating models [45,46]. For example, Hewett [47] developed a spatiotemporal model of collision rates that captures seasonal variations and spatial dependencies across multiple locations. Yang [17] introduced Bayesian dynamic logistic regression to develop a real-time collision risk assessment model, allowing model parameters effectively integrate new data with prior knowledge to dynamically predict risk changes.

In summary, existing studies have proposed various approaches for traffic collision risk prediction, most focus on lane-based environments and static models. The reliance on crash data or predefined behavioral assumptions in simulations limits their responsiveness and generalizability. These methods often lack adaptability to rapidly changing traffic conditions and may not fully capture the complexity of non-lane-based traffic areas. To address these gaps, this study this study aims to develop a data-driven, self-adaptive method for collision risk estimation in complex traffic environments.

## Methodology

### The research framework

**Fig 2:**
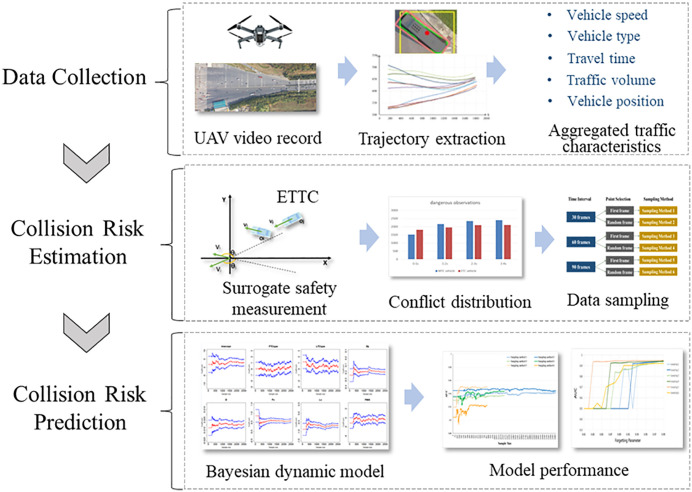
The research framework.

This study proposes a collision risk prediction method tailored for toll plaza diverging areas to explore the dynamic impacts of various factors on vehicle collision risk at a microscopic level. The research framework in Fig 2 outlines the study’s three main components: (1) data collection, involving the recording of vehicle trajectories at the toll plaza diverging area and the extraction of aggregated time series traffic characteristics (see the Data collection and processing section); (2) Vehicle collision risk estimation, which details the two-dimensional surrogate safety measurement (SSM) for estimating collision risk in the non-lane-based area (see the Extended Time-to-Collision section). Also, the sampling strategies for reducing real-time computational burden. (see the Sampling method section); (3) Model development and validation, where a Bayesian dynamic logistic regression model is established to support self-adaptive, real-time conflict prediction (see the Bayesian dynamic logistic regression model section). The model’s performance is assessed through comparative analysis and sensitivity testing (see the Result and discussions section).

### Extended Time-to-Collision

Time-to-collision (TTC) is one of the most widely used surrogate safety measurements (SSMs) for estimating vehicle collision risks. Hayward [48] defined TTC as “the time that remains until a collision between two vehicles would have occurred if the collision course and speed difference are maintained”. TTC can distinguish the unsafe conditions and meanwhile, quantify the severity of vehicle conflicts [49].

The TTC of vehicle j at time step t with the leading vehicle i can be expressed as:


$${TTC}_{j}(t)=\left\{ \begin{array}{c} \frac{X_{i}(t)-X_{j}(t)-L_{i}}{V_{j}(t)-V_{i}(t)},if\;V_{j}(t)>V_{i}(t)\\ \;\infty ,\;if\;V_{j}(t)\leq V_{i}(t) \end{array}\right.\;$$ (1)

where X(t) denotes the vehicle position at time t, V(t) is the vehicle speed at time t, and $L_{i}$ is the length of vehicle i.

**Fig 3:**
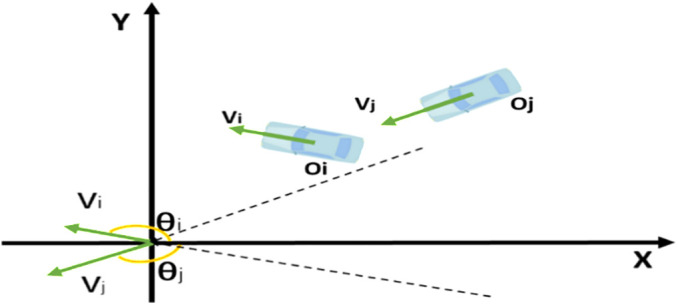
The position of two approaching vehicles in coordinate system.

However, most studies calculated the TTC based on the assumption that the consecutive vehicles are in the same traffic lane or their trajectories cross at a right angle. This assumption would introduce errors in the vehicle collision risk estimation when two vehicles approach to each other at other angles. To overcome this limitation, the Extended Time-to-Collision (ETTC) is applied for calculating vehicle collision risk at the toll plaza diverging area. The ETTC accounts for two-dimensional conflicts, making it more suitable for areas without lane markings [4]. The ETTC is calculated as follows:


$${ETTC}_{j}=-\frac{D_{ij}-\text{0}.\text{5}L_{i}-\text{0}.\text{5}L_{j}}{\frac{\text{1}}{d_{ij}}{\left(O_{i}-O_{j}\right)}^{T}(V_{i}-V_{j})}$$ (2)

where $L_{i}$ and $L_{j}$ are the length of vehicle i and j, respectively; $D_{ij}$ is distance between two vehicles’ centroids; $d_{ij}$ is the distance between two closet points of vehicles; 𝐎 and 𝐕 are two-dimensional coordinates and speed vectors of vehicle’s centroid, as shown in Fig 3. More detailed information about ETTC can be referred in previous studies [4,39]. Since the ETTC is an extension of conventional TTC, the TTC threshold also applies to ETTC. If the ETTC value is lower than the preset threshold, it indicates a potential collision risk between the two vehicles. Previous studies have typically used thresholds ranging from 2 to 4 seconds [50,51], and a threshold of 4 seconds is adopted in this study to capture a broader range of conflict samples.

### Bayesian dynamic logistic regression model

The ordinary logistic regression model can only capture the fixed effects of contributing factors on collision risk at fixed time points using historical data. However, as environmental variables change over time, the effects of these factors also change accordingly. The Bayesian dynamic logistic regression model addresses this issue by using parameters derived from historical data as prior information and updating the model with current data to provide dynamic and accurate results, i.e., it allows for self-adaptive correction of coefficients.

Let Y represent the outcome of vehicle collision risk. Y=1 denotes potential collision risk and Y=0 denotes no potential collision risk. The probability of Y=1 regarding to the influence of explanatory variable *X* in logistic regression model can be expressed as follows:


$$\text{Pr}\left(Y=\text{1}|X\right)=\frac{e^{logit(p)}}{\text{1}+e^{logit(p)}}$$ (3)

$$\text{logit}(\text{p})=\text{ln}\left(\frac{p}{\text{1}-p}\right)=\beta \text{X}+\varepsilon_{n}=\beta_{\text{0}}+\beta_{\text{1}}X_{\text{1}}+\ldots +\beta_{m}X_{m}+\varepsilon_{n}$$ (4)

where β is a vector of estimated coefficients, m is the number of explanatory variables, n is the number of samples, and $\varepsilon_{n}$ is the random error follows a normal distribution with mean zero.

Based on Bayes’ theorem, the Bayesian dynamic logistic regression model introduces time as an additional dimension. It uses parameter information from the previous period as prior information and combines it with current period data to update the parameters. The updating equation of Bayesian dynamic logistic model can be expressed as follows:


$$\text{p}(\theta_{t}|{\text{Y}}^{t})\propto \text{p}(y_{t}|\theta_{t})\text{p}(\theta_{t}|{\text{Y}}^{t-\text{1}})$$ (5)

where ${\text{Y}}^{t}=y_{\text{1}},\ldots ,y_{t-\text{1}},y_{t}$ is all sample data from time step 1 to t, $y_{t}$ is the sample data at current time, ${\text{Y}}^{t-\text{1}}=y_{\text{1}},\ldots ,y_{t-\text{1}}$ is all historical sample data, and $\theta_{t}$ is the parameter to be estimated at time t. This equation demonstrates the updating progress of Bayesian dynamic logistic model. $\text{p}(y_{t}|\theta_{t})$ is the sample information at time t, $\text{p}(\theta_{t}|Y^{t-\text{1}}$is prior information, $\text{p}\left(\theta_{t}|Y^{t}\right)$ is the upgraded posterior information.

Prior information $\text{p}(\theta_{t}|Y^{t-\text{1}})$ mentioned in Equation (5) needs to be recursively estimated through predictive equation. McCormick [52] proposed the equation of state:


$$\theta_{t}=\theta_{t-\text{1}}+\delta_{t}$$ (6)

where $\delta_{t}$ are random vectors obeying independent normal distribution $N(\text{0},W_{t})$, and $W_{t}$ is covariance matrix.

For all the sample data before time t, the recursive estimation begins by supposing:


$$\theta_{t-\text{1}}|{\text{Y}}^{t-\text{1}}\sim N({\hat{\theta }}_{t-\text{1}},{\hat{\Sigma }}_{t-\text{1}})$$ (7)

Then, the prediction equation is


$$\theta_{t}|{\text{Y}}^{t-\text{1}}\sim N({\hat{\theta }}_{t-\text{1}},R_{t})$$ (8)

where $R_{t}=\frac{{\hat{\Sigma }}_{t-\text{1}}}{\lambda_{t}}$. The $\lambda_{t}$ is forgetting parameter, controlling the influence of prior information during each update step, with higher values giving more weight to historical estimates. A value close to 1 (e.g., 0.99) is commonly used to ensure model stability while still allowing adaptation to new data [17]. By combining the posterior information calculated in Equation (8) with the updating equation, the posterior information can be obtained.

When solving the dynamic equations, a key challenge is that the likelihood function of dynamic logistic regression is too complex to derive a closed-form expression for Equation (5). To address this, previous studies have adopted a normal approximation to the right-hand side of Equation (5). This approach is widely used because the dynamic logistic regression model lacks conjugate priors, rendering exact Bayesian updating infeasible. The normal approximation facilitates recursive estimation with manageable computational complexity. Moreover, under regularity conditions and with sufficiently informative priors or large sample sizes, the posterior distribution of logistic regression parameters tends toward normality. As such, this approximation introduces minimal error while preserving the essential properties of the true posterior, making it a theoretically and practically accepted solution in dynamic Bayesian frameworks [52,53].

As mentioned above, ETTC is used to determine whether the vehicle has the collision risk. Thus, the dependent variable ${\text{y}}_{t}$ in Bayesian dynamic logistic regression model can be divided into two cases: (i) ${\text{y}}_{t}=\text{1}$ (has potential collision risk), if the vehicle’s ETTC is below the threshold (ETTC ≤ 4 s); (ii) ${\text{y}}_{t}=\text{0}$ (has no collision risk), if not.

## Data

### Data collection and processing

**Fig 4:**
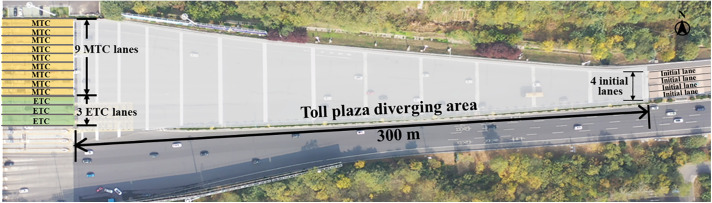
Layout of the diverging area (captured by UAV).

The toll plaza selected for this study is located on the G42 expressway in the northeastern area of Nanjing, China. Fig 4 displays the layout of the toll plaza. The diverging area is 300 meters in length and features three ETC lanes on the left side and nine MTC lanes on the right side at the downstream end. The vehicle trajectories at the toll plaza diverging area were collected using an unmanned aerial vehicle (UAV), which recorded video in 4K ultra-high definition at 30 frames per second (fps). The video data was conducted on March 17th, 2018, a clear and windless day.

Vehicle trajectories were extracted using a video analytics system called the “Automated Roadway Conflicts Identification System (ARCIS),” developed by the University of Central Florida’s Smart and Safe Transportation (UCF SST) team [54]. ARCIS employs the Mask Region-Based Convolutional Neural Network (Mask R-CNN) for video object detection, the Channel and Spatial Reliability Correlation Filters (CSR-CF) algorithm for object tracking, and optical flow for video stabilization to accurately extract trajectories of vehicles with continuous steering or directional changes in non-lane-based traffic areas. In addition, the Savitzky–Golay filtering method and interpolation techniques are further applied for trajectory smoothing and missing data imputation, thereby ensuring the accuracy and completeness of the extracted trajectories. Further details on the video data collection and methodology can be found in our previous study [4](*33*).

The video was recorded for 1.5 hours, of which 50 minutes were selected for extracting vehicle trajectory data. A total of 1,103 vehicle trajectories were tracked, including 1,031 cars, 30 buses, and 42 trucks. Due to the low proportion of buses and trucks, this study only focuses on cars, including 592 MTC cars and 439 ETC cars. The term “vehicles” mentioned in the following text refers to cars. The hourly traffic volume at the study site ranged from 1,050–1,740 vehicles per hour (calculated as 6 times of the 10-min volume intervals). The trajectory data includes information on time ID and vehicle position. Additional parameters, such as vehicle speed, travel direction, the vehicles’ initial lanes and final toll lanes, and traffic flow, can be derived through further calculations.

**Fig 5:**
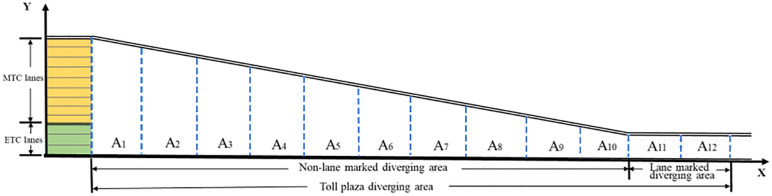
Layout of diverging area sub-segments.

To better quantify the impact of the surrounding environment on vehicle collision risk prediction, the diverging area is divided into 12 sub-segments. As shown in Fig 5, each sub-segment is 30 meters long and sequentially numbered from 1 to 12. Sub-segment 1–10 are in the non-lane marked area, and the 11–12 are in the lane-marked area. The aggregated traffic characteristics that may affect the collision risk at toll plaza diverging area are listed in Table 1. The characteristics were selected based on the conflicts between the subject vehicle and its leader, and thus include information of the leading and following vehicles. In addition to capturing vehicle kinematics, the characteristics also account for specific factors of toll plaza diverging areas, such as the toll collection types and the absence of lane markings. These aggregated traffic characteristics are designed to reflect key behavioral and environmental elements influencing collision risk in non-lane-based toll plaza diverging areas.

**Table 1:** Aggregated traffic characteristics.

Category	Variable	Description
Variables of the leading vehicle	${LTC}_{type}$	An indicator variable for leading vehicle’s toll collection types, 0 for a MTC vehicle, 1 for an ETC vehicle
	$L_{v}$	The leading vehicle’s speed, m/s.
Variables of the following vehicle	D	Vehicle’s travel distance, the distance after vehicle entering the diverging area (m)
	T *	Vehicle’s travel time, the time after following vehicle entering the diverging area (s)
	${FTC}_{type}$	An indicator variable for toll collection types of the following vehicle, 0 for a MTC vehicle, 1 for an ETC vehicle.
	$F_{v}$	The following vehicle’s speed
Other variables	$D_{ij}$	The distance between two vehicles’ centroids
	FMIX	The mix measure of MTC and ETC vehicles in the following vehicle’s current sub-segment
	FVO*	The average traffic volume in the following vehicle’s current sub-segment, vph.
	${FP}_{ETC}$*	The percentage of ETC vehicles in the following vehicle’s current sub-segment
	LM*	An indicator variable for lane marking features of the following vehicle location, LM = 0 for non-lane marked, LM = 1for lane marked.

Before developing the Bayesian dynamic logistic regression model, Pearson correlation tests were conducted for all independent variables. Four variables were found to be significantly associated with other variables (P < 0.05, |r| > 0.5), namely T, FVO, and ${FTC}_{type}$. As shown in Table 1, these variables marked with * were excluded from both the standard and dynamic Bayesian logistic regression models. The full Pearson correlation matrix is provided in Appendix A for reference.

### Sampling method

With the ETTC threshold set at 4 seconds, a total of 75,732 observations were obtained, including 16,368 risky samples and 59,364 safe samples. To clearly present the distribution of risky samples, the threshold was divided into one-second intervals, and the distributions for risky samples of ETC and MTC vehicles within each interval are shown in Table 2. Smaller ETTC values indicate higher levels of collision risks. The number and percentage of risky samples involving MTC vehicles are higher than those for ETC vehicles, likely due to more frequent weaving and lane-changing behaviors among MTC vehicles. For both ETC and MTC vehicles, the number of risky samples decreases as the conflict severity increases, consistent with the typical distribution pattern of severe vehicle conflicts. Additionally, ETC vehicles generally travel at higher speeds, which may explain why ETC vehicles account for a larger proportion of the 0–1 s interval, while the percentage of MTC vehicles is higher in other time intervals.

**Table 2:** The description of dangerous samples.

Time Interval	Dangerous Samples				Total	
	MTC vehicle		ETC vehicle			
	Number	Percentage	Number	Percentage	Number	Percentage
0-1 s	1515	9.26%	1811	11.06%	3326	20.32%
1-2 s	2156	13.17%	1954	11.94%	4110	25.11%
2-3 s	2344	14.32%	2090	12.77%	4434	27.09%
3-4 s	2393	14.62%	2105	12.86%	4498	27.48%
Total	8408	51.37%	7960	48.63%	16368	100.00%

Ideally, video data collection should be performed at the frame level, meaning that continuous data can be obtained at t = 1, 2,…T, where t is the data for each frame and T reprents all data. The model would perform better if all continuous data were used for estimation. However, due to computational constraints, it is not feasible to use the entire continuous dataset in practical applications. To address this, an interval sampling method is employed to obtain discrete data for reducing the computational burden.

**Fig 6:**
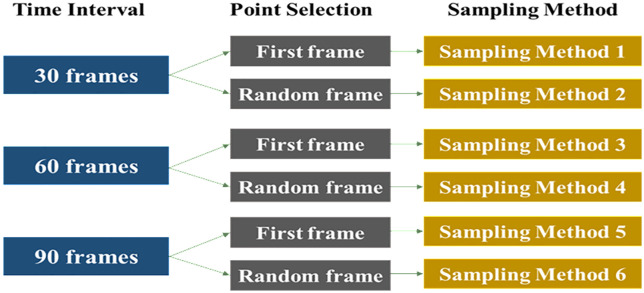
Illustration of six sampling methods.

To avoid the impact of the sampling method on model results, we designed several sampling strategies for testing the Bayesian dynamic logistic regression models. Specifically, we defined three time intervals: 30 frames, 60 frames, and 90 frames. Since the UAV video provides 30 frames per second, these sampling methods extract one frame every 1 second, 2 seconds, and 3 seconds, respectively. Additionally, two sampling strategies were employed for selecting sample points within each time interval: (1) selecting the first frame in each interval, and (2) randomly selecting one frame from each interval. This results in a total of six discrete datasets, derived from six different sampling methods (three time intervals multiplied by two selection strategies), as shown in Fig 6.

All sampling points were sorted in ascending order of frame time for the updating process. Frames with smaller numbers are treated as prior information, while frames with larger numbers represent posterior data. Table 3 presents the statistics of risky samples across the different sampling methods. The overall collision rate in the dataset is 22.27%. The collision rates for samples obtained using the six sampling methods are 22.23%, 22.58%, 22.11%, 21.63%, 23.31%, and 21.64%, respectively. The ANOVA analysis indicates that there is no significant difference in the proportion of risky samples among the various sampling methods.

**Table 3:** The statistics of dangerous samples among different sampling methods.

Sampling method	Method 1	Method 2	Method 3	Method 4	Method 5	Method 6
Percentage of risky samples	22.23%	22.58%	22.11%	21.63%	23.31%	21.64%
sample size	2,524	2,524	1,262	1,262	8,41	8,41

It should be noted that while the interval sampling method may result in the loss of some data, it enhances real-time operability and provides a feasible solution for the model’s real-time self-adaptive updates. For each current discrete sampling point in the Bayesian dynamic logistic regression model, the estimation results from all previous sampling points are used as prior information and incorporated into the posterior model. This allows the model to dynamically update prior information, leading to more effective and accurate predictions.

## Result and discussions

**Fig 7:**
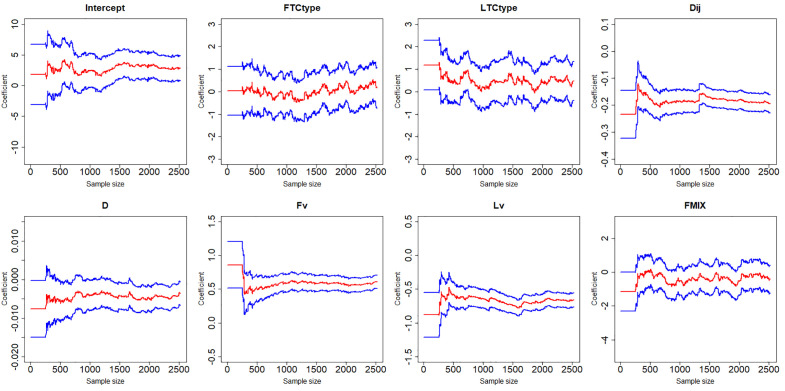
Updating coefficients in Bayesian dynamic logistic regression model of sampling method 1.

**Fig 8:**
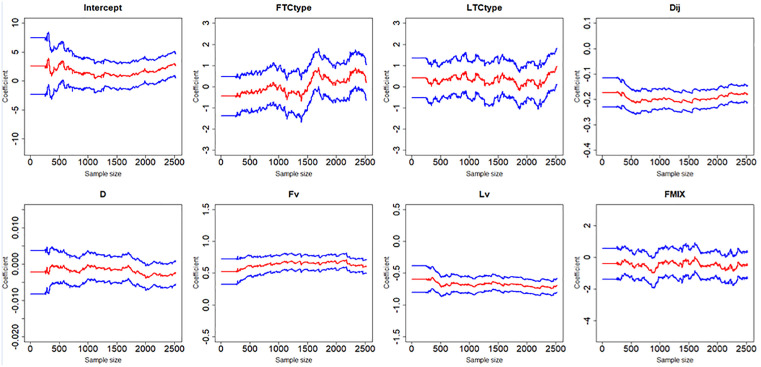
Updating coefficients in Bayesian dynamic model of sampling method 2.

**Fig 9:**
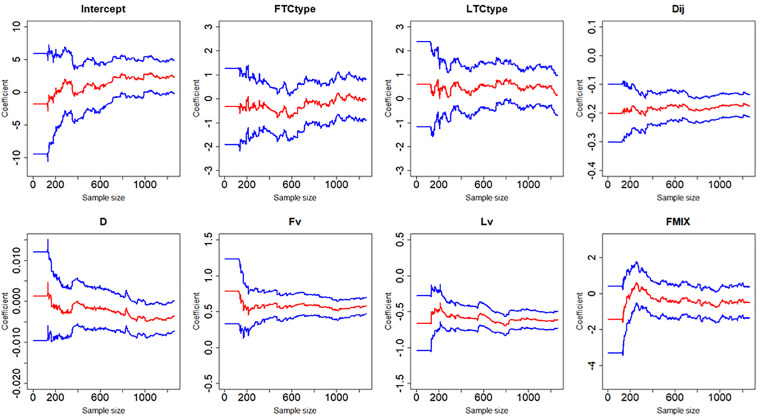
Updating coefficients in Bayesian dynamic model of sampling method 3.

**Fig 10:**
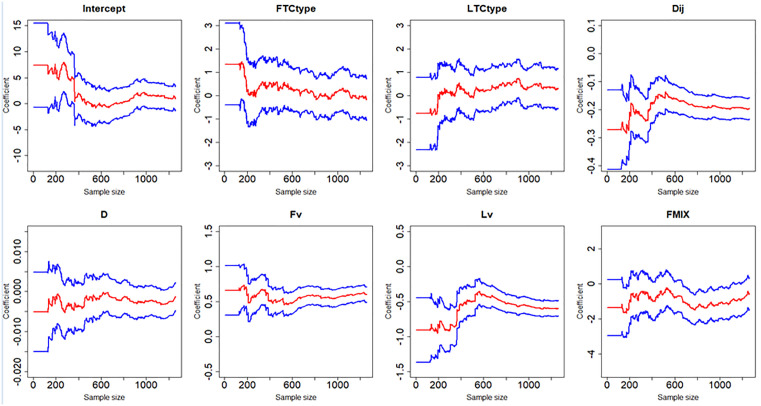
Updating coefficients in Bayesian dynamic model of sampling method 4.

**Fig 11:**
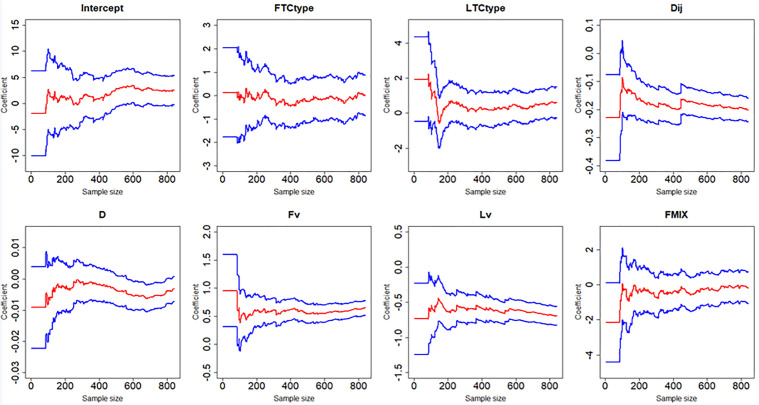
Updating coefficients in Bayesian dynamic model of sampling method 5.

**Fig 12:**
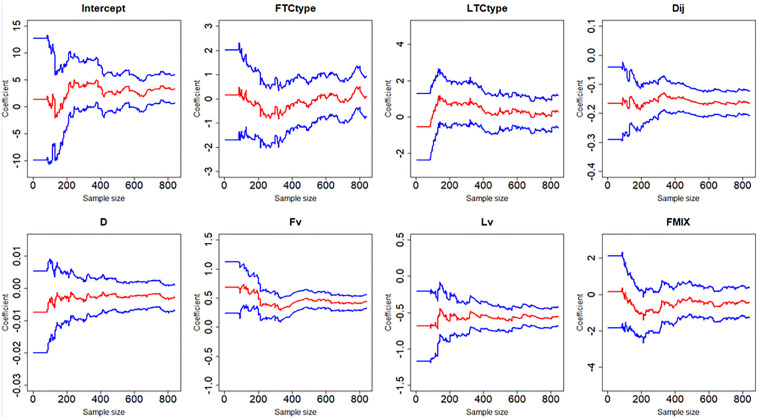
Updating coefficients in Bayesian dynamic model of sampling method 6.

Figs 7–12 display the dynamic curves of parameter self-adaptive correction in the Bayesian dynamic logistic regression model under six different sampling methods. The x-axis represents the sample size, which increases over time as more data are collected. The y-axis shows the coefficient values of all independent variables, including the intercept. The red line represents the estimated mean coefficient, and the blue lines represent the results within two standard deviations above and below the mean. The forgetting parameter $\lambda_{t}$ is set to 0.99, and sensitivity analysis of this value will be conducted later.

From the dynamic estimation results, the coefficients of all independent variables dynamically change over time. Although the six different sampling methods yield distinct discrete data, their updated parameters show consistent trends. For all sampling methods, the coefficients of variables related to vehicle dynamics, such as D, $L_{v}$, $F_{v}$ and $D_{ij}$, exhibit consistent signs, indicating more consistent and predictable influences on collision risk. This suggests that traffic management strategies should prioritize maintaining safe spacing and speed control of vehicles in diverging areas to mitigate collision risk. In contrast, the coefficients of ${FTC}_{type}$, ${LTC}_{type}$ and FMIX show more substantial fluctuations, with both positive and negative values. This may be because the value of ${FTC}_{type}$, ${LTC}_{type}$ and FMIX are determined by the toll collection types of vehicles entering the toll plaza diverging area. Their effects are more sensitive to the changes of traffic flow composition. This implies that operational strategies targeting lane configuration or toll system design may require ongoing monitoring and periodic reassessment based on accumulated data. It should be noted that some coefficient values fluctuate across the zero line, which demonstrates that factors have different effects on vehicle collision risk under different traffic conditions, even opposite sometimes.

**Fig 13:**
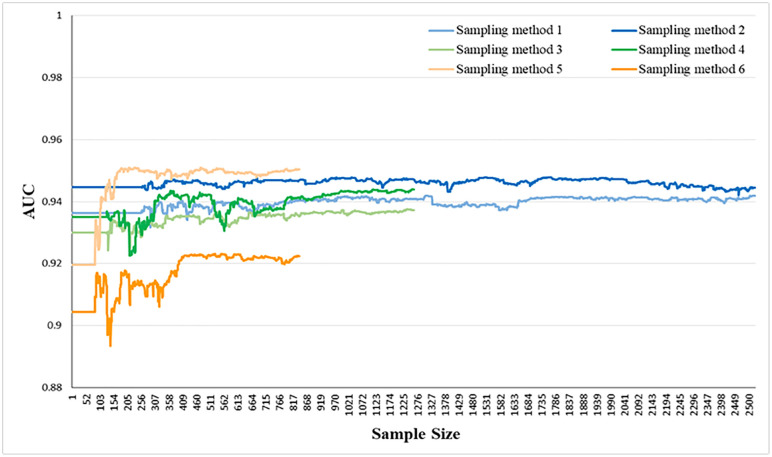
The AUC values of different sampling methods in the cumulative sample size.

The updating trends of AUC values for Bayesian dynamic logistic regression models using different sampling methods are shown in Fig 13. As the sample size increases, the AUC values gradually rise and stabilize, with only minor fluctuations. All six methods achieve AUC values above 0.9, indicating good predictive performance and demonstrating the model’s self-adaptive updating capability. The AUC values of sampling methods 1–5 converge to approximately 0.94, while method 6 stabilizes at a slightly lower value around 0.92. Sampling methods with shorter intervals tend to produce more stable AUC values throughout the updating process. Overall, the results show that while different sampling strategies may influence the final AUC values to a limited extent, the proposed model remains robust and effective across all tested sampling methods.

The final results of Bayesian dynamic logistic regression model based on six different sampling methods are listed in Table 4. The indicator area under receiver operating characteristics curve (AUC) is employed to comprehensively assess the model evaluate accuracy in this study. The AUC takes values from 0 to 1, and a larger AUC value indicates a better model performance. All the Bayesian dynamic logistic regression models perform well as their AUC values are more than 0.9. The seven variables all have significant effects on vehicle collision risk in Bayesian dynamic models. The coefficients of ${FTC}_{type}$, ${LTC}_{type}$ and FMIX differ greatly among different sampling methods, and their corresponding standard errors are relatively larger. The coefficients of ${FTC}_{type}$ in sampling 3 and 4 are even opposite with other sampling methods. While coefficients of D, $L_{v}$, $F_{v}$, $D_{ij}$ and standard errors show consistent trends among different sampling methods. Vehicle conflicts are more likely to occur in the upstream of diverging area. A lower mixed degree of ETC and MTC vehicles, larger distance between two vehicles would decrease the vehicle collision risk.

**Table 4:** Results of Bayesian dynamic models with different sampling methods.

Sampling method	AUC	Statistical indicator	Intercept	D	$F_{v}$,	$L_{v}$	$D_{ij}$	${LTC}_{type}$	${FTC}_{type}$	FMIX
Sampling method 1	0.942	Coefficients	2.827*	−0.004*	0.606*	−0.662*	−0.195*	0.464*	0.171*	−0.422*
		Std	1.014	0.002	0.049	0.053	0.017	0.433	0.452	0.413
Sampling method 2	0.944	Coefficients	2.634*	−0.002*	0.602*	−0.696*	−0.180*	0.957*	0.202*	−0.500*
		Std	1.040	0.002	0.053	0.054	0.016	0.427	0.424	0.412
Sampling method 3	0.938	Coefficients	2.300*	−0.004*	0.580*	−0.618*	−0.175*	0.144*	−0.046*	−0.489*
		Std	1.260	0.002	0.058	0.059	0.019	0.418	0.427	0.431
Sampling method 4	0.944	Coefficients	0.996*	−0.001*	0.599*	−0.598*	−0.195*	0.311*	−0.142*	−0.581*
		Std	1.162	0.002	0.055	0.055	0.019	0.427	0.440	0.452
Sampling method 5	0.950	Coefficients	2.545*	−0.003*	0.647*	−0.695*	−0.202*	0.609*	0.006*	−0.178*
		Std	1.410	0.002	0.065	0.066	0.021	0.447	0.432	0.447
Sampling method 6	0.922	Coefficients	3.314*	−0.003*	0.442*	−0.556*	−0.166*	0.292*	0.095*	−0.444*
		Std	1.321	0.002	0.060	0.065	0.021	0.440	0.411	0.413

*Significant at the 95% confidence level.

In addition, standard logistic regression models were established based on six sampling methods for comparison, as shown in Table 5. The AUC values of Bayesian dynamic logistic regression models and standard logistic regression models with different time interval data sampling methods are all more than 0.9, indicating good predictive performance for both models. While there is no substantial difference in the mean coefficient values between the two models, more factors have significant effects on vehicle collision risk in the Bayesian dynamic logistic regression models. In particular, variables related to toll collection types, such as ${FTC}_{type}$, ${LTC}_{type}$ and FMIX, exhibit stronger and more consistent significance in the Bayesian dynamic models, suggesting that they better capture the characteristics of toll plaza environments. Variables $L_{v}$ and $F_{v}$ show more stable significance in the Bayesian dynamic models, reflecting their robustness in capturing vehicle interactions. These differences demonstrate that the Bayesian dynamic model not only improves predictive performance but also offers clearer interpretability by identifying more influential factors. In addition, compared with standard logistic regression model, the Bayesian dynamic logistic regression model requires only 20% of data during initialization and can continuously update its estimates as the sample size increases, significantly reducing the demand for computing resources in collision risk prediction.

**Table 5:** Model results of ordinary logistic regression model.

Sampling method	AUC	Intercept	D	$F_{v}$,	$L_{v}$	$D_{ij}$	${LTC}_{type}$	${FTC}_{type}$	FMIX
Sampling method 1	0.942	2.806*	−0.004*	0.611*	−0.697*	−0.192*	0.426*	/	−0.446*
Sampling method 2	0.948	1.761*	−0.002*	0.637*	−0.682*	−0.193*	0.378*	/	−0.454*
Sampling method 3	0.937	2.110*	−0.004*	0.579*	−0.629*	−0.183*	/	/	−0.479*
Sampling method 4	0.944	0.563*	/	0.635*	−0.612*	−0.201*	0.336*	/	−0.814*
Sampling method 5	0.950	2.340*	−0.004*	0.677*	−0.728*	−0.209*	0.497*	/	/
Sampling method 6	0.922	2.996*	/	0.461*	−0.575*	−0.166*	/	/	−0.477*

*Significant at the 95% confidence level

**Fig 14:**
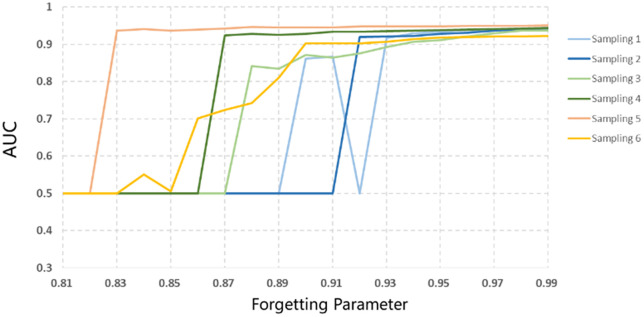
Sensitivity analysis for forgetting parameter$\lambda_{t}$.

In Bayesian dynamic logistic regression models, the forgetting parameter $\lambda_{t}$ plays an important role in determining the model’s reliance on prior information during estimation. Thus, a sensitivity analysis was conducted for $\lambda_{t}$ values ranging from 0.80 to 1.00. As shown in Fig 14, the forgetting parameter of different sampling methods shows similar variation trends. When $\lambda_{t}$ is less than 0.82, the AUC values remain around 0.5. With the $\lambda_{t}$ increases to 0.93, the AUC values gradually rise to around 0.9. The best AUC (0.95) is achieved when $\lambda_{t}$ is close to 0.99.

The sensitivity analysis demonstrates that the relationship between the AUC and forgetting parameter in Bayesian dynamic logistic regression models is not strictly monotonic. A smaller forgetting parameter indicates less prior information is used in updating process, so that the past data have less effects on current model estimation. Insufficient prior information caused by small forgetting parameter leads to a poor model performance. Increasing the forgetting parameter enriches the prior information, thereby enhancing the model’s prediction accuracy. Based on these results, a forgetting parameter in the range of 0.98 to 0.99 is recommended for practical applications, as it provides a good balance between responsiveness to new data and retention of useful prior information. This range consistently yields the highest AUC values across different sampling strategies, indicating robust model performance.

## Conclusion

A Bayesian dynamic logistic regression approach is developed in this study for predicting vehicle collision risks in toll plaza diverging areas. By incorporating a surrogate safety measure suitable for non-lane-based traffic environments, extracting aggregated traffic features, and designing discrete sampling strategies, the approach utilizes the self-adaptive parameter updating capability of dynamic Bayesian modeling to provide timely and adaptive conflict predictions. The main conclusions of this study are as follows:

(1) The Bayesian dynamic logistic regression model demonstrated superior interpretability and computational efficiency compared to standard logistic regression. It identified more significant influencing factors of vehicle collision risk and required only 20% of the data during initialization, while continuously updating its estimates as new data arrived.
(2) As the volume of data increases, the AUC values of Bayesian dynamic logistic regression models based on different sampling methods consistently increase, all exceeding 0.9, demonstrating its robust self-adaptive correction capability and high predictive performance.
(3) Vehicle conflicts are more likely to occur in the upstream of the diverging area. A lower mixed proportion of ETC and MTC vehicles, as well as a larger distance between two vehicles, reduces the risk of conflicts. The sensitivity analysis results for the forgetting parameter $\lambda_{t}$ indicate that richer prior information improves the model’s predictive accuracy.

The Bayesian dynamic logistic regression model based on sampling aggregated traffic characteristics in this study significantly enhances prediction efficiency while capturing the continuous dynamic changes in traffic conditions. It is particularly well-suited for processing current traffic data, characterized by large volumes and high generation speeds. In China, as toll lane configurations and payment methods at toll plazas undergo upgrades, changing from historical data-based vehicle collision risk prediction models to dynamically updating models can support the development of real-time traffic conflict warning systems at toll plaza diverging areas. By continuously capturing changes in traffic flow composition, it enables dynamic adjustment of upstream lane guidance strategies and timely optimization of ETC and MTC lane allocations. These applications can help reduce vehicle weaving and merging conflicts in diverging areas, thereby enhancing overall traffic safety.

Future research could further extend this study in two directions. First, applying the proposed approach to toll plazas with different geometric and operational characteristics would help assess its generalizability and scalability across diverse traffic environments. Second, incorporating additional influencing factors, such as weather conditions and vehicle types, could enhance the model’s ability to capture contextual variations in collision risk and improve its practical applicability.

## Supporting information

S1 File Appendix A. Pearson correlation matrix of input variables.
(DOCX)

S2 File Vehicle trajectory dataset of the toll plaza diverging area. (XLSX)
