# Supplementary material for: Predicting microscopic vehicle collision risks at toll plaza diverging area using bayesian dynamic logistic regressions
Source: PLoS One. 2025 Oct 27;20(10):e0332929. doi: 10.1371/journal.pone.0332929 (PMC12558522; doi:10.1371/journal.pone.0332929)
Supplement: S1 File — Pearson correlation matrix of input variables. (DOCX) [file pone.0332929.s001.docx]

**Appendix A. Pearson Correlation Matrix of Input Variables**

| Variables | $T$ | $D$ | ${FTC}_{type}$ | $F_{v}$ | ${LTC}_{type}$ | $L_{v}$ | ${FP}_{ETC}$ | $FMIX$ | FVO | $D_{ij}$ | $LM$ |
| --- | --- | --- | --- | --- | --- | --- | --- | --- | --- | --- | --- |
| $T$ | 1.000 |  |  |  |  |  |  |  |  |  |  |
| $D$ | 0.959* | 1.000 |  |  |  |  |  |  |  |  |  |
| ${FTC}_{type}$ | -0.044 | -0.005 | 1.000 |  |  |  |  |  |  |  |  |
| $F_{v}$ | -0.536* | -0.396 | 0.114 | 1.000 |  |  |  |  |  |  |  |
| ${LTC}_{type}$ | -0.022 | -0.022 | 0.092 | -0.012 | 1.000 |  |  |  |  |  |  |
| $L_{v}$ | -0.500* | -0.427 | -0.001 | 0.208 | 0.150 | 1.000 |  |  |  |  |  |
| ${FP}_{ETC}$ | -0.045 | -0.013 | 0.720* | 0.091 | 0.425 | 0.042 | 1.000 |  |  |  |  |
| $FMIX$ | 0.009 | -0.012 | 0.070 | -0.032 | 0.087 | 0.081 | 0.113 | 1.000 |  |  |  |
| FVO | -0.226 | -0.210 | 0.072 | 0.160 | -0.009 | 0.166 | 0.025 | 0.505* | 1.000 |  |  |
| $D_{ij}$ | 0.011 | 0.047 | -0.010 | 0.077 | -0.043 | -0.162 | -0.003 | -0.198 | -0.344 | 1.000 |  |
| $LM$ | -0.506* | -0.548* | 0.001 | 0.098 | 0.004 | 0.202 | -0.000 | -0.030 | 0.138 | -0.017 | 1.000 |

* |r| > 0.5
